# Percutaneous closure of an ultra-long-tunnel-type patent foramen ovale: a rare case with multimodal imaging guidance

**DOI:** 10.1186/s12872-026-05529-x

**Published:** 2026-02-03

**Authors:** Bo Li, Ming Li, Beibei Song

**Affiliations:** 1 Zibo Central Hospital, West Campus, No. 10 Shanghai Road, Zhangdian District, Zibo City, Shandong Province China; 2 Binzhou Medical University Second Clinical Medical College (Yantai Affiliated Hospital)-Zibo Central Hospital, No. 10 Shanghai Road, Zhangdian District, Zibo City, Shandong Province China

**Keywords:** Patent foramen ovale, Right heart contrast echocardiography, Ultra-long-tunnel, Multimodal imaging, Percutaneous closure

## Abstract

Patent foramen ovale (PFO) is a common congenital cardiac anomaly, and ultra-long tunnel PFO (> 20 mm) presents a significant interventional challenge despite routine standard closure. A 68-year-old female with recurrent headaches, chest pain history, and prior right coronary artery stenting was confirmed to have a 37-mm ultra-long tunnel PFO via transesophageal echocardiography (TEE) and digital subtraction angiography (DSA). After initial percutaneous closure failure, the patient was successfully treated with a modified trans-tunnel puncture technique and deployment of an Abbott 18/25 mm Amplatzer PFO Occluder. Follow-up showed gradual resolution of residual shunt, with no shunt detected by June 2025. The imaging-guided modified trans-tunnel puncture technique is an effective strategy for complex ultra-long tunnel PFOs.

**Supplementary Information:**

The online version contains supplementary material available at 10.1186/s12872-026-05529-x.

## Clinical presentation

A 68-year-old female presented with a chief complaint of “paroxysmal chest discomfort for 10 months and aggravated headache for 1 month.”

### Timeline of illness:10 months prior

 The patient developed exertional chest tightness. 1 month prior: Coronary angiography at a local hospital revealed proximal occlusion of the right coronary artery. Two stents were implanted (PCI). Following this, she took dual antiplatelet therapy regularly. Current Admission: While the chest pain resolved post-PCI, she experienced recurrent headaches.

### Physical examination

 Vital signs were stable (BP 121/73 mmHg, HR 88 bpm). Physical examination was unremarkable: the heart rhythm was sinus with no pathological murmurs, the consistent intensity of the first heart sound and the absence of pulse deficits (heart rate equal to pulse rate), and the cardiac boundary was normal. Neurological examination showed no focal deficits.

## Past medical history

The patient underwent a right nephrectomy 7 years ago and lumbar spine surgeries 20 and 10 years ago. She denied hypertension, diabetes, or a family history of cardiac disease.

## Differential diagnosis

### The patient presented with two distinct sets of symptoms

The paroxysmal, exertional nature of the chest pain, which resolved after stenting, confirmed Coronary Artery Disease (CAD) as the cause. Headache and Cerebral Ischemia: The recurrent headaches and multiple ischemic spots on MRI were found. With sinus rhythm confirmed (ruling out atrial fibrillation), the PFO with a massive right-to-left shunt was identified as the etiology for the cerebral ischemic events and headaches.

## Examination

### Echocardiography

**Fig. 1:**
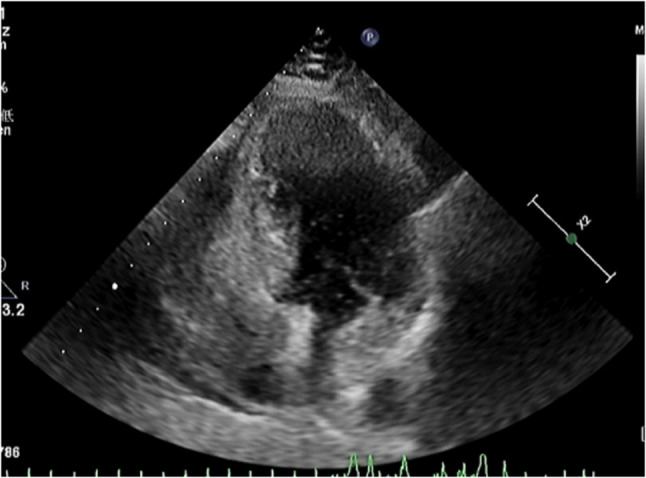
Routine preoperative right heart contrast echocardiography for the patient’s PFO closure procedure revealed a large number of microbubbles in the left heart

**Fig. 2:**
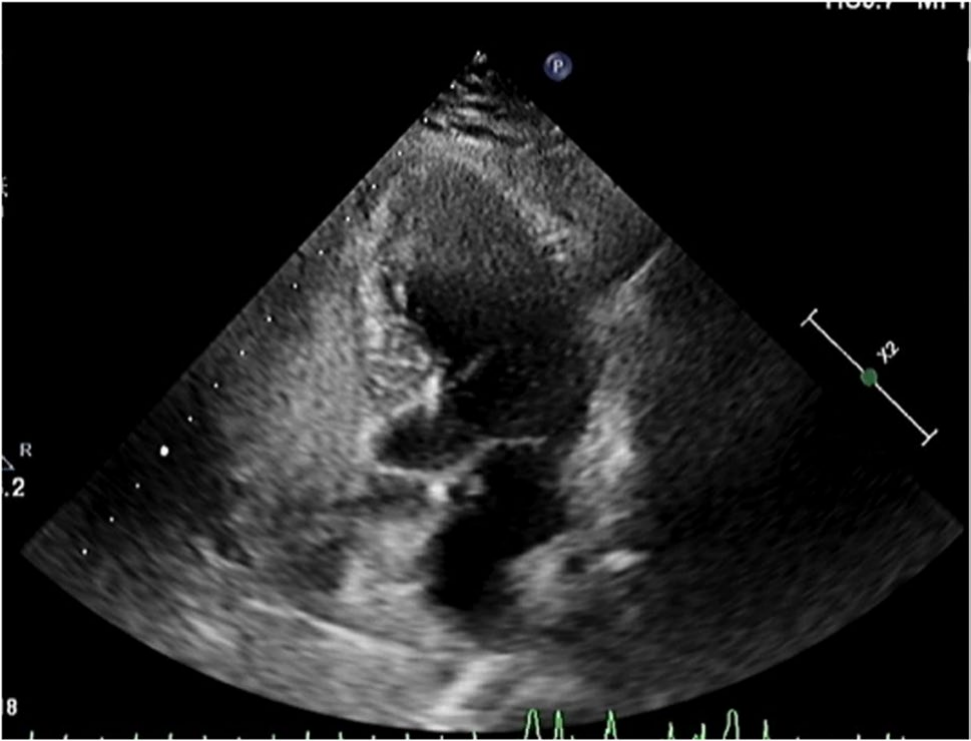
After the Valsalva maneuver, the number of microbubbles in the left heart increased significantly

**Fig. 3:**
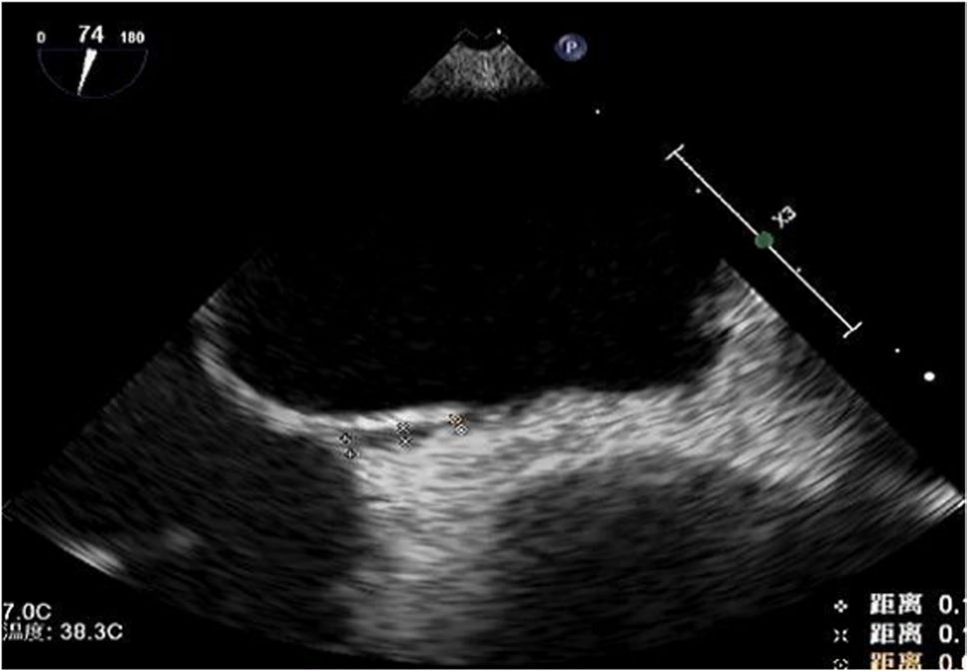
Transesophageal echocardiography shows the right atrial separation of the patent foramen ovale detected at the fossa ovalis in the middle of the interatrial septum

**Fig. 4:**
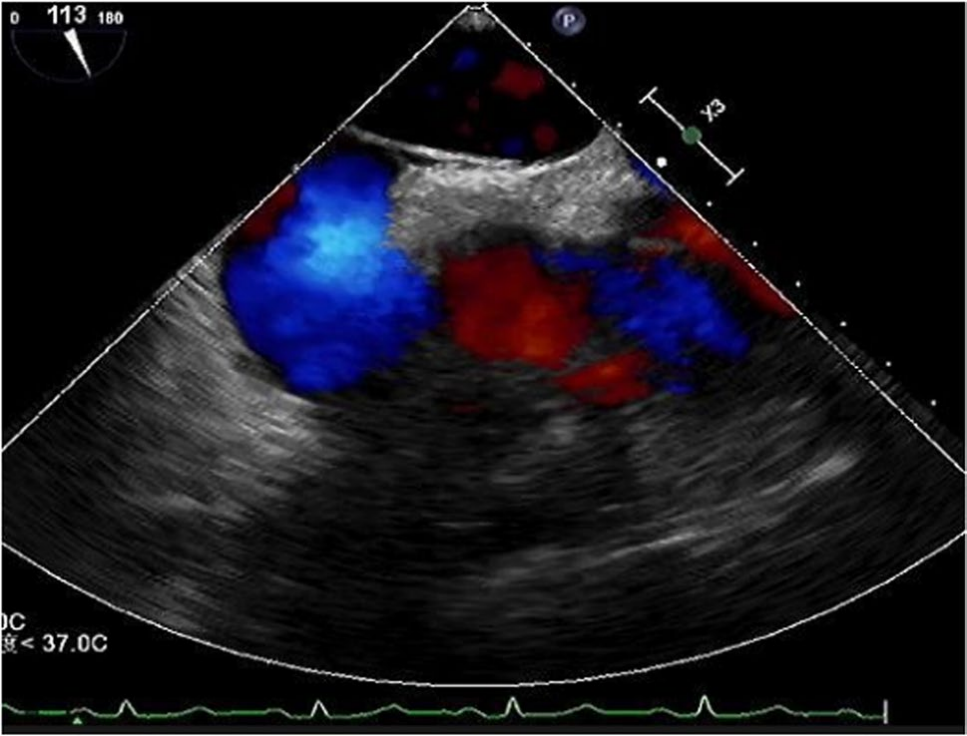
Transesophageal echocardiography (TEE) revealed a PFO

 Preoperative right heart contrast echocardiography revealed a massive right-to-left shunt (RLS) during the Valsalva maneuver (Figs. 1 and 2). Transesophageal echocardiography (TEE) demonstrated a significant separation of the septal leaflets. (Figures 3 and 4).

### Imaging examination

**Fig. 5:**
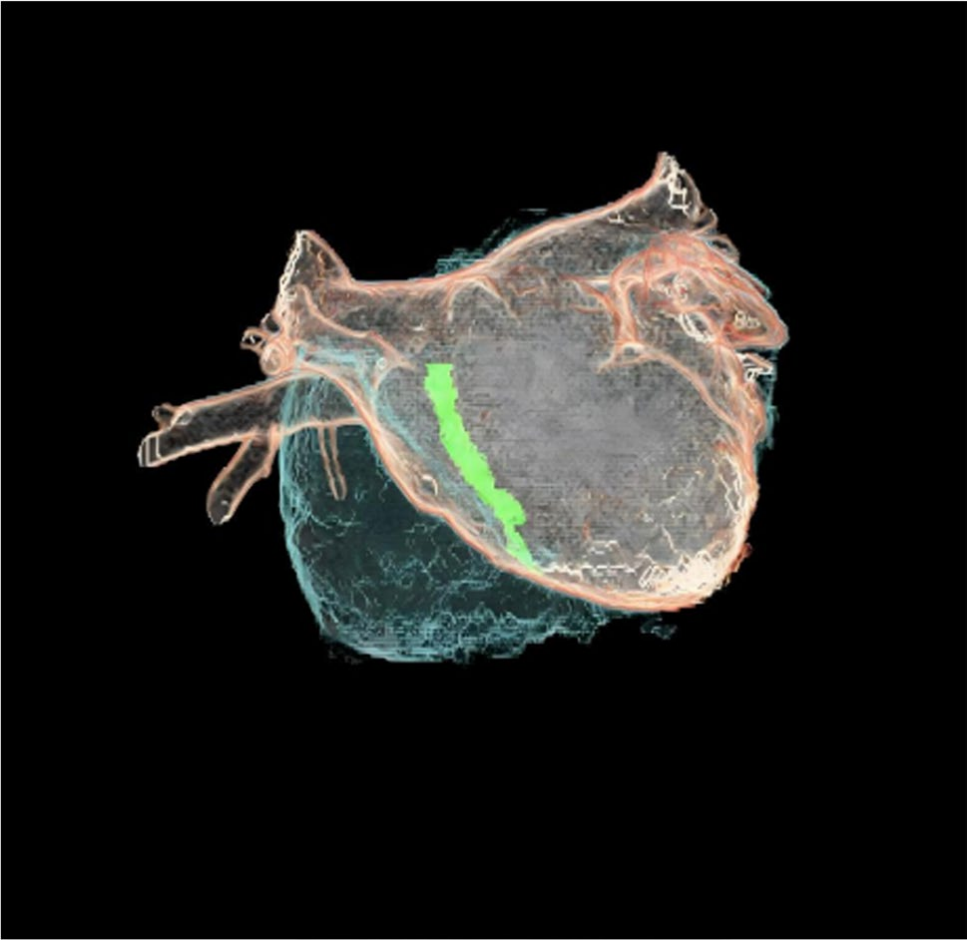
CT atrial reconstruction revealed the anatomical relationship between the PFO and the atria

Although CT atrial reconstruction is not a routine method for PFO evaluation, it was utilized in this specific case to visualize the complex 3D anatomy of the ultra-long tunnel (Fig. 5).

Brain MRI identified multiple acute-to-subacute infarcts in the left cerebellum, basal ganglia, and frontal lobes.

**Fig. 6:**
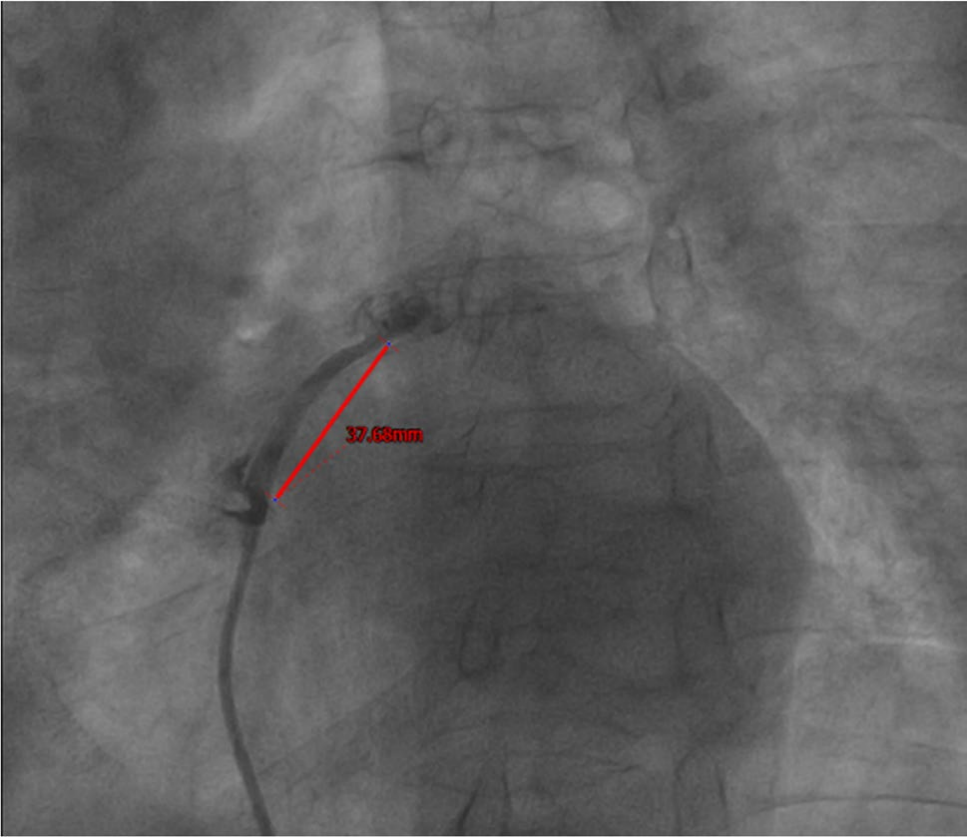
DSA during the PFO closure procedure showed an ultra-long tunnel with a length of 37 mm

**Fig. 7:**
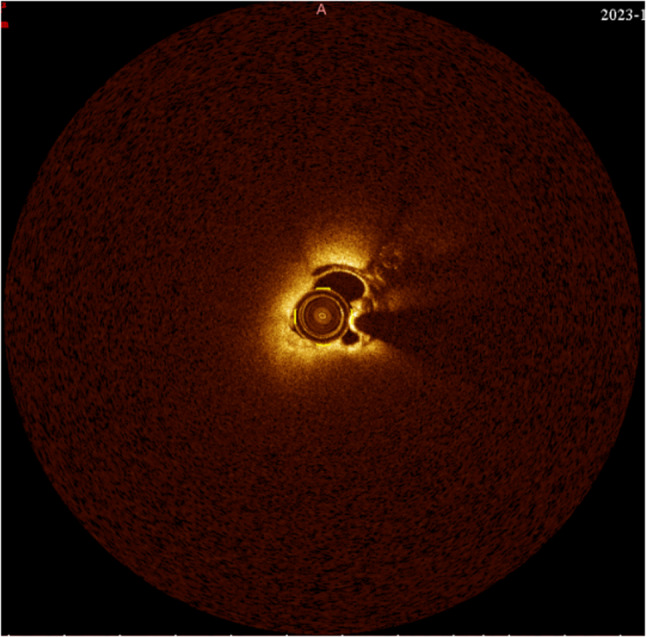
OCT revealed rough walls of the PFO tunnel, visible thrombus-like echoes, and a tunnel length of 37 mm

Intraoperative Imaging: Intraoperative digital subtraction angiography (DSA) and Optical Coherence Tomography (OCT) confirmed the PFO tunnel length to be 37 mm (Figs. 6 and 7) and revealed rough tunnel walls, highlighting the risk of in-tunnel thrombosis [1, 2].

## Treatment

**Fig. 8:**
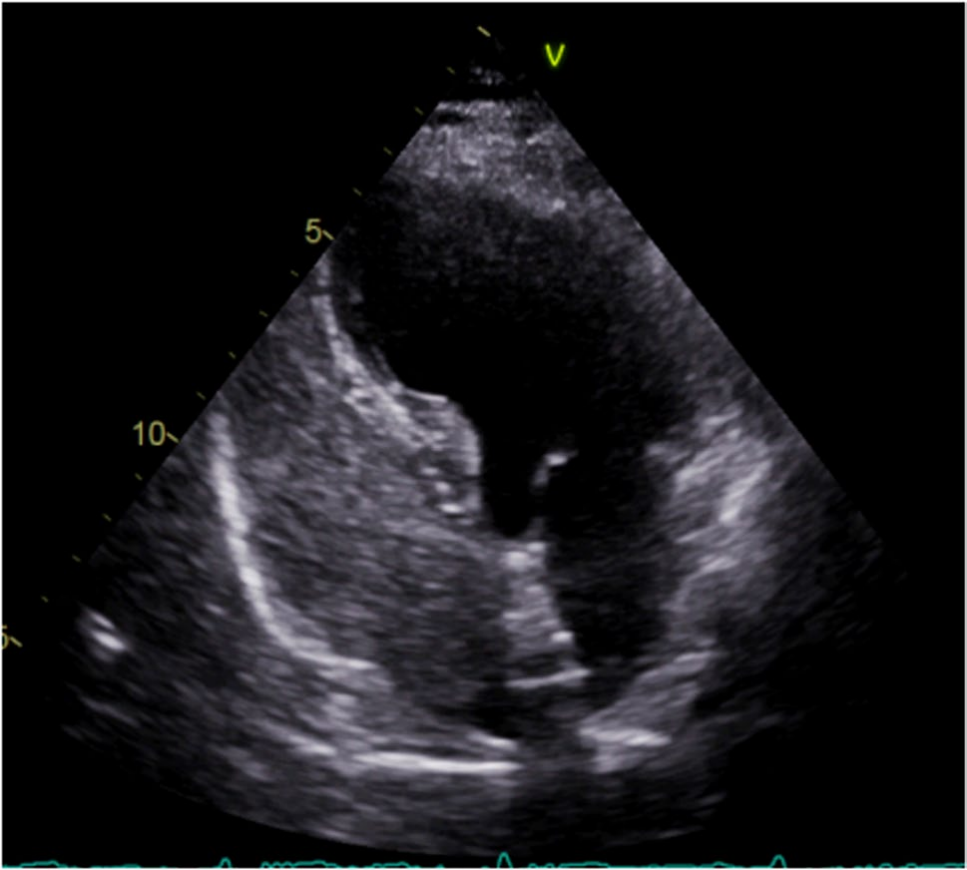
The patient`s the latest right heart contrast echocardiography showed no microbubbles

The patient was initially treated with antiplatelet therapy. First Intervention Attempt (Failed): Standard percutaneous closure was attempted. However, the 37-mm tunnel length exceeded the waist length of the occluder. The right atrial disc was constrained inside the tunnel and unable to exit into the right atrium. The first operattion was failed. Second Intervention Attempt (Successful): A modified trans-tunnel single-wall puncture technique was employed using a 9 F delivery sheath and an Abbott 18/25 mm Amplatzer PFO Occluder.The delivery sheath was advanced through the right atrial ostium (secundum) of the PFO and positioned approximately halfway into the tunnel. Instead of traversing the full length of the tunnel, the tip of transseptal sheath was used to puncture the septum primum (tunnel wall) directly from within the tunnel to access the left atrium. Deployment: The 25-mm the left atrial (LA) of the occluder was released and gently pulled to snug up against the center of the atrial septum. Subsequently, the 18-mm Right Atrial (RA) disc was released and the occluder was fully deployed by using the two discs to pinch the two layers of the septum together. Mechanism: This configuration allowed the two discs to pinch the septal layers together slightly. The RA disc completely covered the natural right atrial opening, sealing the entry point. Although the opening of the PFO on the left atrial side was not covered by the occluder, the device completely eliminated the patency of the tunnel. Outcomes and Follow-up: The procedure was successful. Contrast echo showed a moderate shunt (< 30 bubbles) after the first month of the operation. Contrast echo showed a trivial shunt after 12 Months (2024). Repeat examination showed no microbubbles in June 2025 (Fig. 8), confirming complete endothelialization and closure.

## Discussion

1. Anatomical Challenge
  This case involves a 37-mm ultra-long tunnel, far exceeding the typical definition of a “long tunnel” (≥ 8–10 mm) [3, 4]. This anatomy results from the excessive overlap of the septum primum and secundum due to failed fusion [5]. In such cases, standard closure fails because the device becomes “stuck” within the tunnel.
2. Technique and Safety (Addressing Septostomy Risks)
  Standard atrial septostomy involves creating a new hole away from the PFO. Literature (e.g., observations by Ilka et al.) warns that if a device covers a septostomy site but fails to cover the original PFO edge, the risk of embolism persists. Our trans-tunnel puncture technique specifically mitigates this risk. By entering through the right atrial ostium and puncturing the wall internally: we created a secure point in the septum primum for the LA disc. Crucially, the Right Atrial Disc is deployed directly over the original PFO ostium.Therefore, the “right atrial PFO edge” is completely sealed by the device [6].
3. AF Exclusion
  Given the patient’s age (68), atrial fibrillation (AF) was a differential diagnosis. While long-term monitoring was not performed, ECGs during hospitalization consistently showed sinus rhythm. The combination of confirmed sinus rhythm and high-risk PFO anatomy strongly supports the PFO as the reason of stroke [7].

## Conclusion

For ultra-long tunnel PFOs where standard crossing fails, the trans-tunnel single-wall puncture technique is a safe and effective strategy. It ensures device stability and, importantly, guarantees sealing of the right atrial PFO inlet, preventing residual shunting.

## Supplementary Information

Supplementary Material 1.

Supplementary Material 2.

Supplementary Material 3.

Supplementary Material 4.

## Data Availability

No datasets were generated or analysed during the current study.
