# Supplementary material for: Percutaneous closure of an ultra-long-tunnel-type patent foramen ovale: a rare case with multimodal imaging guidance
Source: BMC Cardiovasc Disord. 2026 Feb 3;26:191. doi: 10.1186/s12872-026-05529-x (PMC12954885; doi:10.1186/s12872-026-05529-x)
Supplement: Supplementary file 3 — Supplementary Material 3. [file 12872_2026_5529_MOESM3_ESM.docx]

1. Funding Declaration

This case report did not receive any funding from public, commercial, or non-commercial funding agencies. The corresponding declaration has been added to the manuscript, and the content is:Funding Declaration: This research received no specific grant from any funding agency in the public, commercial, or not-for-profit sectors.

2. Ethics, Consent to Participate, and Consent to Publish Declarations

This manuscript is a retrospective case report based on the clinical diagnosis and treatment data of a single patient. During the patient's hospitalization and treatment, our medical team has strictly abided by the relevant medical ethics regulations. Before collecting the patient's clinical data and using it for this case report, we have obtained the patient's written informed consent for both "participation in the clinical data collection for medical research" and "publication of the case-related information (including clinical data, imaging materials, etc.) in academic journals".

Consent to Publish: Written informed consent for publication of this case report (including clinical details, imaging findings, and other relevant information) was obtained from the patient, and the patient has confirmed that the published content will not involve her personal privacy (such as name, ID number, etc.).
