# Supplementary figures and images for: Percutaneous closure of an ultra-long-tunnel-type patent foramen ovale: a rare case with multimodal imaging guidance
Source: BMC Cardiovasc Disord. 2026 Feb 3;26:191. doi: 10.1186/s12872-026-05529-x (PMC12954885; doi:10.1186/s12872-026-05529-x)

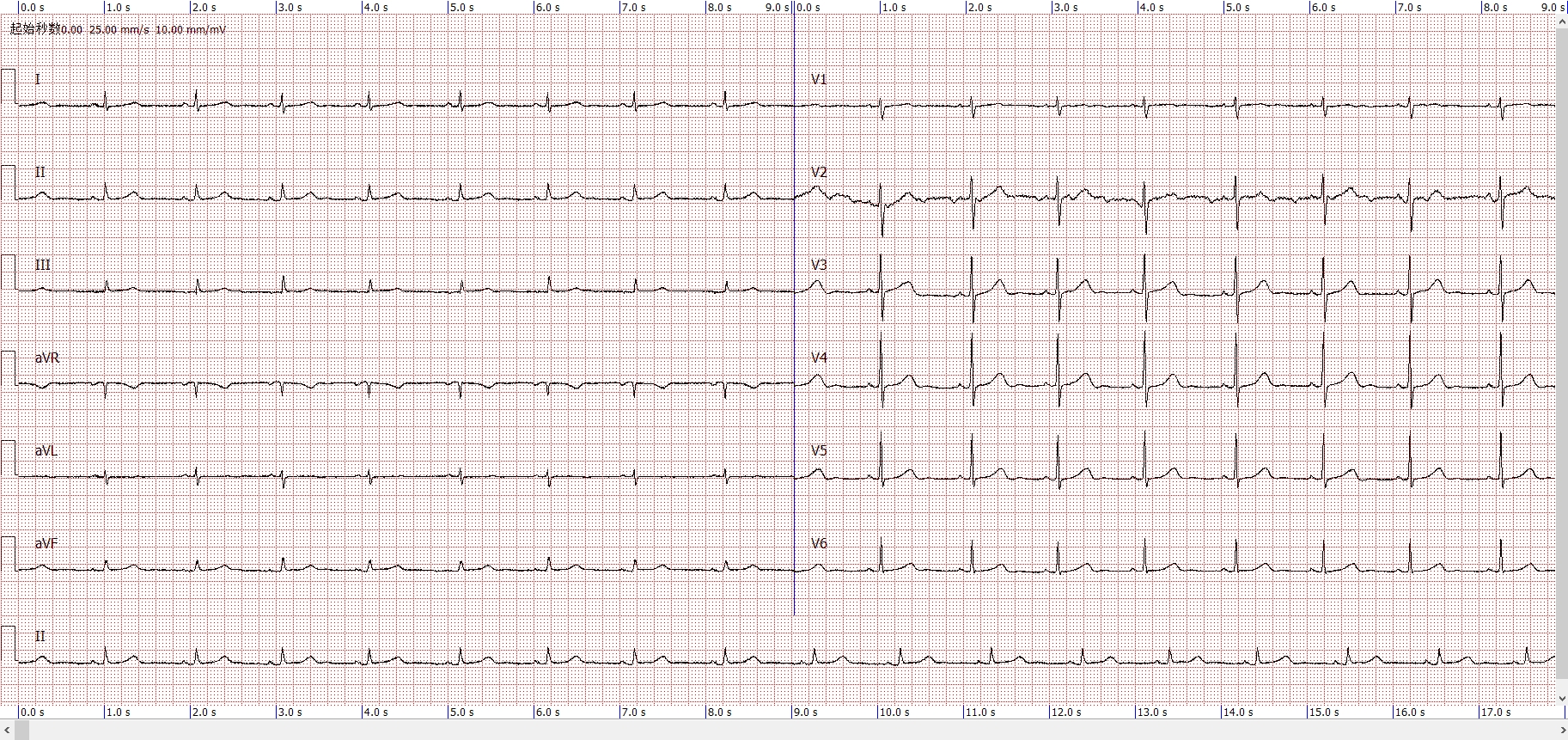

Supplement: Supplementary file 4 — Supplementary Material 4. [file 12872_2026_5529_MOESM4_ESM.zip › submit ECG/2017.10.18..jpeg]

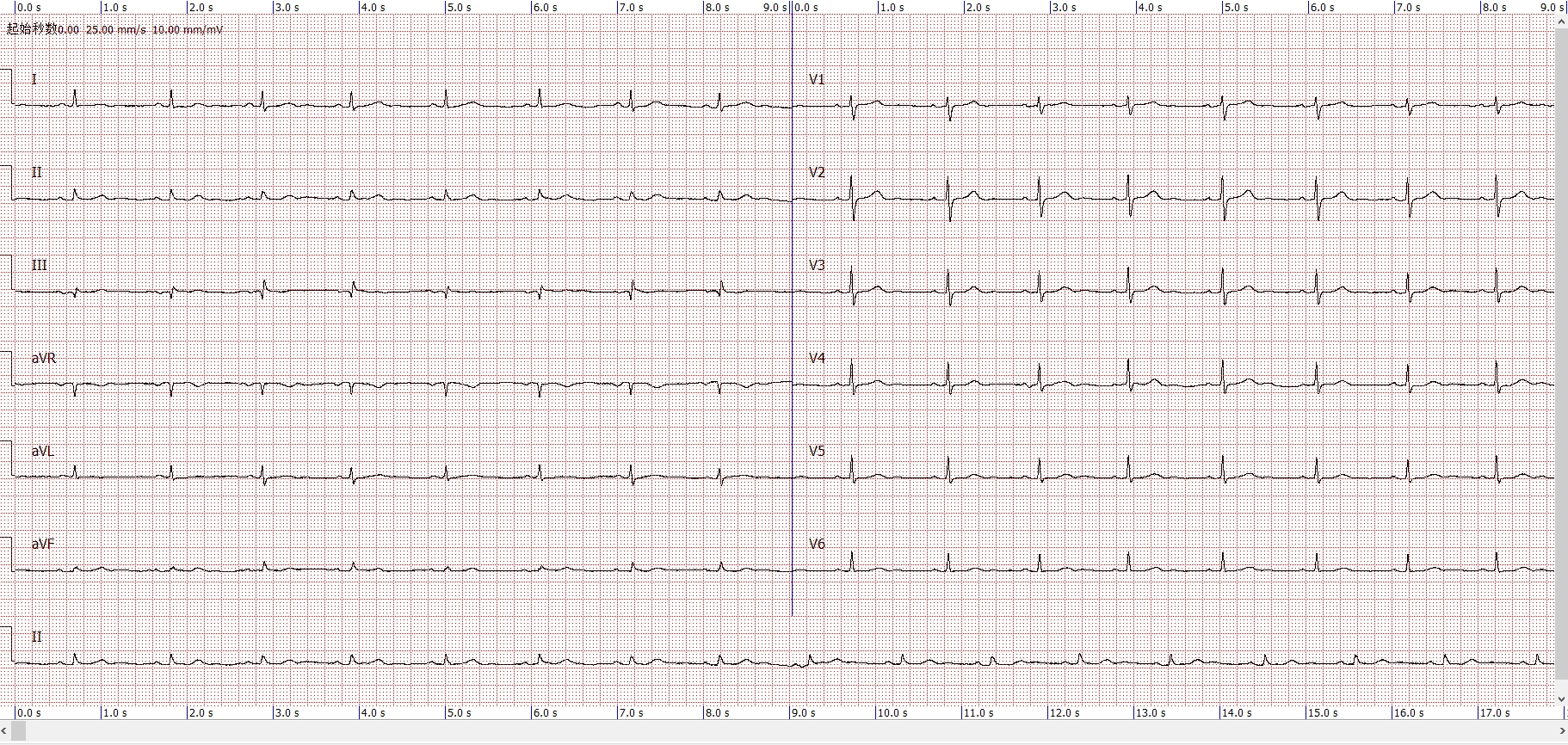

Supplement: Supplementary file 4 — Supplementary Material 4. [file 12872_2026_5529_MOESM4_ESM.zip › submit ECG/2023.09.04..jpeg]

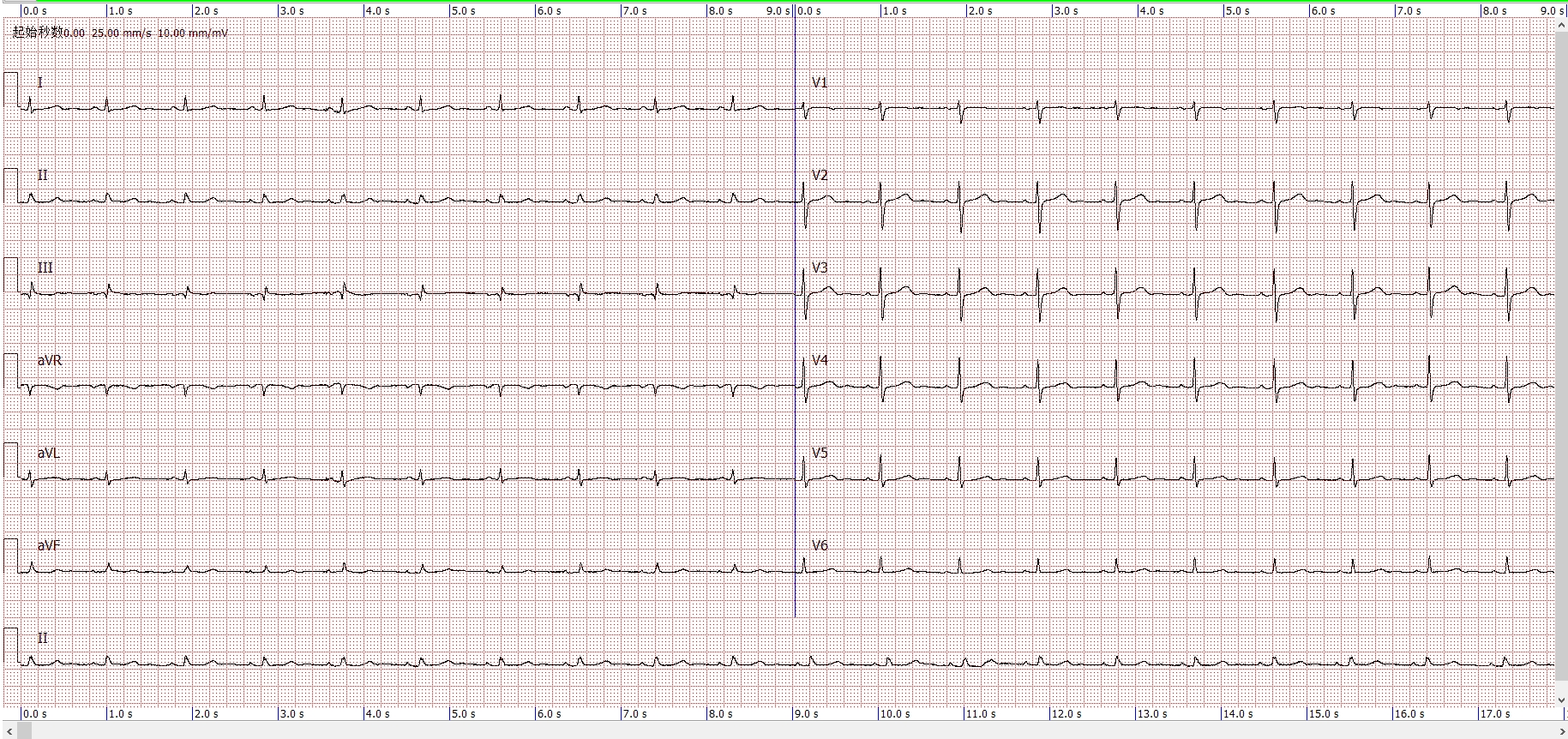

Supplement: Supplementary file 4 — Supplementary Material 4. [file 12872_2026_5529_MOESM4_ESM.zip › submit ECG/2023.10.20..jpeg]

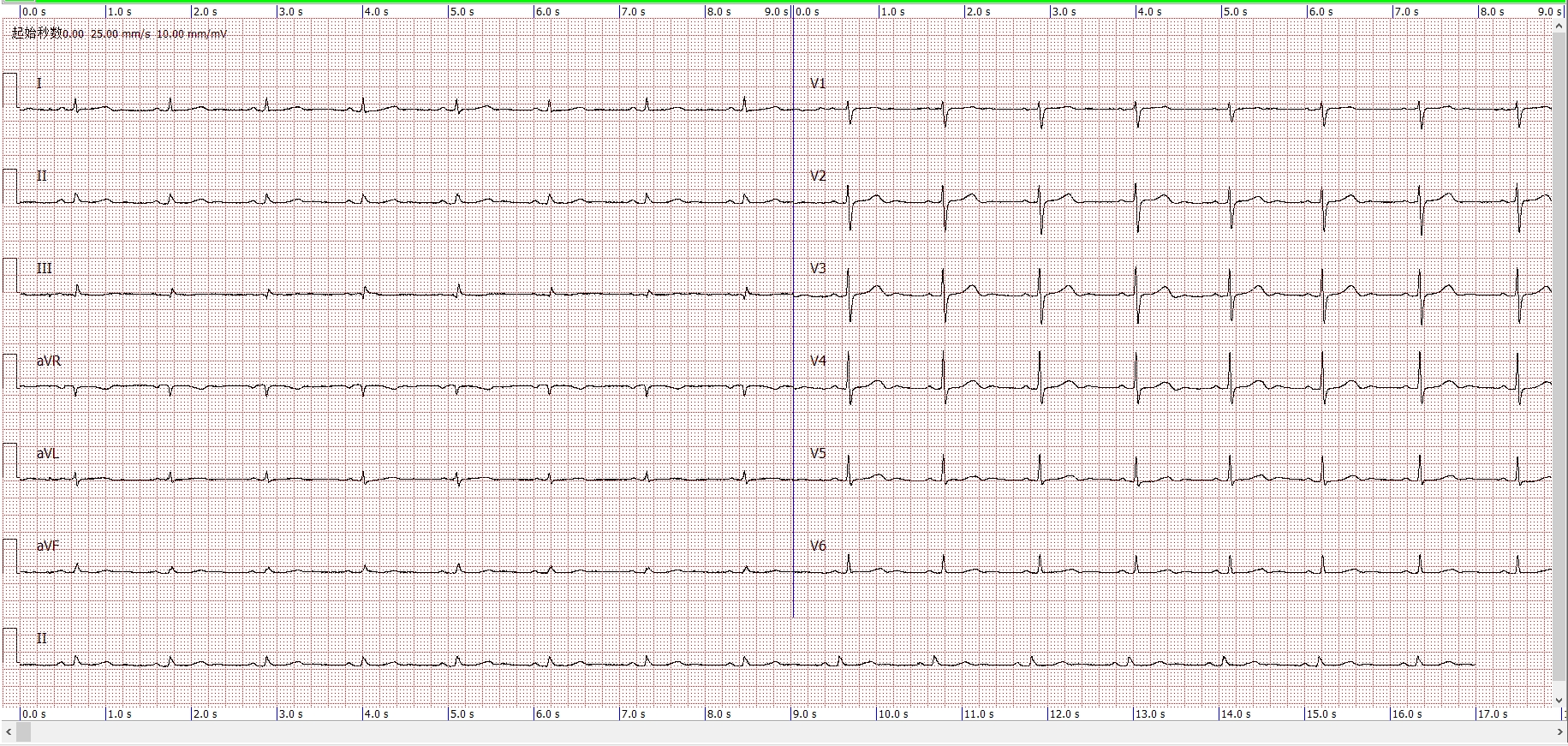

Supplement: Supplementary file 4 — Supplementary Material 4. [file 12872_2026_5529_MOESM4_ESM.zip › submit ECG/2023.10.21..jpeg]
